# Electric Vehicle Charging Station Location towards Sustainable Cities

**DOI:** 10.3390/ijerph17082785

**Published:** 2020-04-17

**Authors:** Xiangyu Luo, Rui Qiu

**Affiliations:** 1 Business School, Sichuan University, Chengdu 610064, China; xiangyu_l2019@163.com; 2 Uncertainty Decision-Making Laboratory, Sichuan University, Chengdu 610064, China

**Keywords:** sustainable cities, sustainable transport, electric vehicles, charging station location, reservation service, healthier lives

## Abstract

Electric vehicles, a significant part of sustainable transport, are attracting increasing attention with the development of sustainable cities. However, as supporting facilities of electric vehicles, public charging stations are of great significance to the promotion of electric vehicles. This paper proposes an electric vehicle charging station location model to improve the resource utilization of electric vehicles for sustainable cities. In this model, reservation services, idle rates during off-peak periods, and waiting time during peak periods are considered. Finally, a case from Chengdu, China, is used to examine the effectiveness of the proposed model. Then, further analyses of reservation ratios and penetration rates are conducted. The results show that the introduction of a reservation service has a positive effect on reducing the total cost, which would provide further support for sustainable cities and have an even greater impact on healthier lives.

## 1. Introduction

The rapid speed of urbanization leads to many environmental problems [1]. The sustainable development of cities has become an increasing concern in recent years [2]. One of the goals of the United Nations (UN) 2030 agenda for sustainable development is “Sustainable cities and communities”, aiming to make cities and human settlements safe, resilient, and sustainable [3]. Sustainability in cities involves using technological innovations and knowledge from various scientific fields for ensuring urban residents’ quality of life that can be sustained over a long period [4]. A sustainable city is defined as one in which people and businesses continuously endeavor to improve their natural, built, and cultural environment [5]. In other words, sustainability in cities aims to sustain the quality of life for urban residents while a sustainable city is envisaged as a balance of natural, built, and cultural elements. Sustainable transport is a significant part of sustainable cities [6]. Greenhouse gas (GHG) emissions are concentrated in urban areas [7], and carbon dioxide emitted by vehicles accounts for about 40% of the total urban carbon emissions [8]. Meanwhile, persistent pollutants, heavy metals, and particulate matter produced by transportation have adverse effects on human health and are becoming some major concerns for people who live and work in urban areas [9,10,11]. Consequently, the introduction of more environmentally sustainable alternative transport solutions is essential [12].

Electric vehicles (EVs) have great potential to increase energy efficiency, reduce greenhouse gas emissions, and diversify energy resources for more sustainable transport [13,14,15]. Therefore, promoting the development of EVs is critical to deal with climate change challenges and achieve sustainable transport [16,17]. As an important guarantee for the development of EVs, the development level of electric vehicle charging stations (EVCS) has a direct impact on the development speed and quality of the electric vehicle industry [18,19,20]. However, the shortage of EVCS has become one of the important factors hindering the development of the electric vehicle industry [21]. Consequently, finding reasonable locations of EVCS has become a key issue to promote the development of the electric vehicle industry.

**Figure 1:**
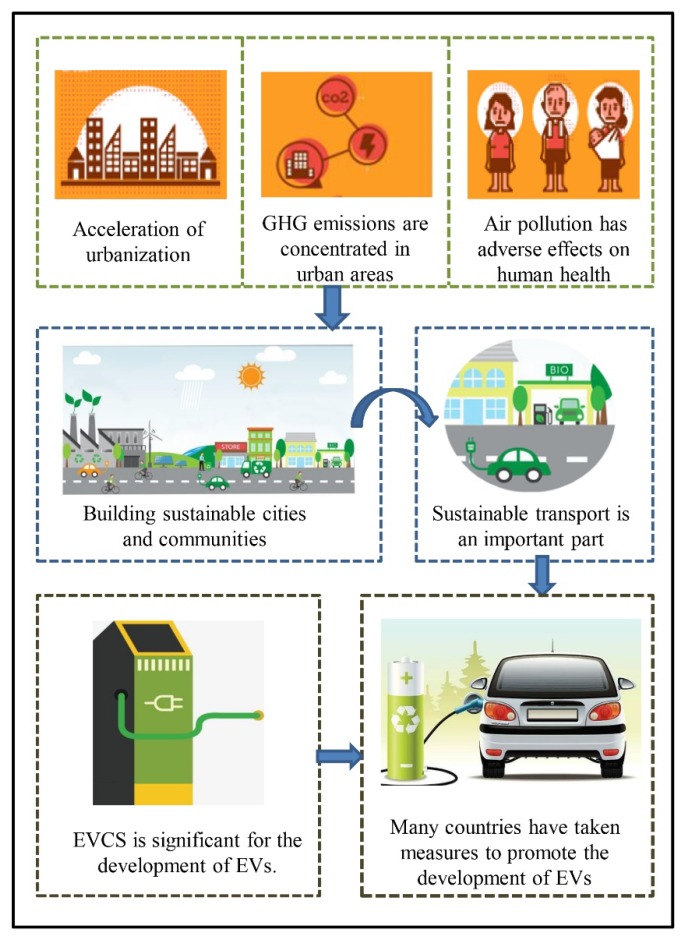
The relationship between electric vehicle charging stations (EVCS) and sustainable cities.

Smart cities, which rely on the deployment of information and communication technology [22], provide a new opportunity for the development of EVs that can be applied for sustainability in cities [23]. Smart cities emphasize the effective integration and utilization of resources for sustainable development [24]. As an important part of EV planning in smart cities, EVCS is crucial for the development of sustainable cities. Figure 1 describes the relationship between EVCS and sustainable cities. With the increase in the scale of use of EVs, the optimal location of EVCS has become a hot topic and focus of research. Many scholars have analyzed and provided solutions to the EVCS location from different perspectives and have achieved many objectives, mainly based on the following topics: (1) the coverage problem [25,26,27,28,29,30], (2) the heuristic electric vehicle charging placement [31,32,33], (3) the flow-capturing approach [34,35,36,37,38,39,40], and (4) the traffic network equilibrium problem [41,42,43,44,45]. These works provide a lot of valuable references for us to explore the EVCS location problem. The purpose of charging stations is to maximize the net social benefit in some countries (e.g., China) as EVs are now in the promotional phase for these countries [25,28,29]. Long charging queues result in inconvenience and high social cost [46]. With the rapid development of the Internet, a reservation service has become popular as it allows consumers to choose suitable charging stations and times according to their demand, avoiding the long waiting time and enhancing customer convenience. In other words, a reservation service can not only improve system efficiency, but also increase customer satisfaction, promoting the development of the industry. Therefore, this paper aims to minimize the total social cost of EVCS location planning, allowing for the impact of the reservation service, and seeks to find the optimal locations of charging stations, providing a reference for sustainable urban planning and development.

In this paper, an EVCS location model, which is based on location theory involving the maximal covering location problem (MCLP) and queuing theory, is proposed to improve the resource utilization of electric vehicles for sustainable cities [47,48]. Then, the genetic algorithm (GA) is applied to solve this model. Moreover, analyses of results obtained are performed to provide implications and recommendations. These analyses provide insights into the effects of a reservation service and tolerable waiting time on the total social cost of the EVCS locations. The remainder of this paper is organized as follows. Section 2 first provides a formal description of the EVCS location and then proposes a model for the EVCS location. In Section 3, a case from Chengdu, China is given to show the practicality and effectiveness of the proposed model, and the results are computed by the GA. Section 4 contains further analysis and discussions. Finally, the conclusions, limitations of the study, and future directions are presented in Section 5.

## 2. Materials and Methods

### 2.1. Problem Description

EVs, with zero pollutant emissions and low noise, are regarded as a new energy transportation tool [49]. Therefore, to promote sustainable urban transport, EVs are being promoted worldwide as the future means of transportation because of their potential environmental benefits [50]. For example, in China, the government has introduced many preferential policies to promote the development of the EV industry. However, the insufficient and unreasonable construction of EVCS is still an important factor hindering its development. Currently, EVs are in the promotional phase in China, and the purpose of charging stations is to maximize the net social benefit by minimizing the social cost [25,28,29]. Therefore, this paper aims at minimizing the total social cost of EVCS location planning from the perspective of the government.

### 2.2. Assumptions

**Figure 2:**
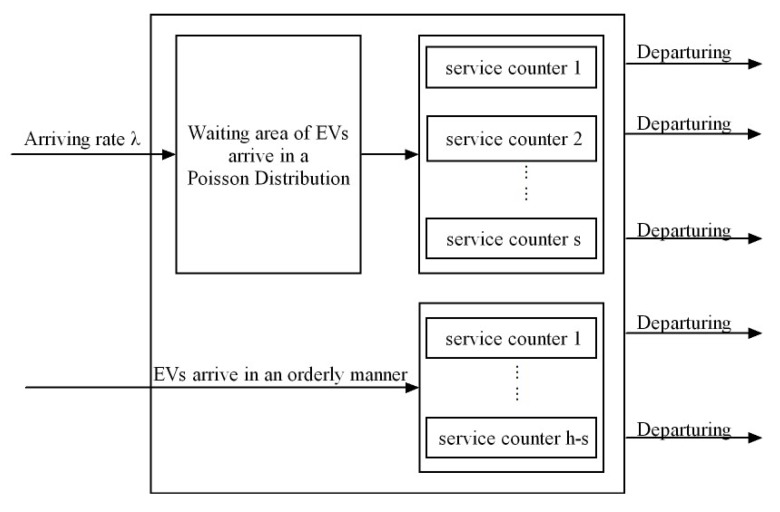
The queuing system of EVCS.

This model is constructed based on the MCLP [47]. The model can be stated as: if there is a station, EVs within the coverage charging radius can be charged. Unreasonable location of charging stations will cause congestion and idleness [25]. Some studies have focused on the service capacity, such as covering as many EVs as possible within the budget, without considering congestion. There are also some studies that only restrict the waiting time and fail to consider the fact that a large number of charging piles may be lying idle. Therefore, we introduce the queuing system to describe the service process and improve resource utilization through average waiting time and average idle rate constraints. Usually, the shorter notation A/B/c can be used to describe a queueing system, where A is the interarrival time distribution, B is the service time distribution, and c is the number of servers [48]. In this study, interarrival and service times are assumed to follow an exponential distribution with c identical servers. Then the queueing system is an M/M/c queuing system. Meanwhile, the purpose of the introduction of the reservation service is to avoid waiting in the queue, but in reality, there may be the problem of a user with a reservation not arriving on time. Therefore, we appropriately reduce the service rate of charging piles that provide a reservation service to provide a buffer time for the users, so that the users can arrive in an orderly manner and do not need to wait in the queue. Since range anxiety that leads to full recharging is also a significant factor affecting charging behavior [51], we assume drivers leave the charging station at a 100% charge to avoid range anxiety. To facilitate the presentation of the essential ideas without losing generality, the following basic assumptions are made in this paper:

(1) Each EV can be charged at one of the charging stations that cover it.

(2) Each charging station is modeled as an M/M/s queuing system, following the principle of first come first served.

(3) EV drivers will give priority to the reservation service and the reserved EVs will arrive in an orderly manner. The queuing system is shown in Figure 2.

(4) EV drivers leave the charging station at a 100% charge.

### 2.3. Notations

The mathematical notations used in this study are listed in Table 1.

### 2.4. Model Formulation

#### 2.4.1. Demand Estimating

State of charge (SOC), electricity consumption rate, and battery capacity have significant impacts on the EV drivers’ charging behavior [52,53] and can determine how many times a day the driver needs to recharge. The relationship between them can be expressed as:

(1) $$\sigma =\frac{(L_{soc}-S_{soc})E}{\beta d_{average}}.$$

where *L_soc_* denotes the SOC when drivers leave the EVCS, *S_soc_* denotes the SOC when drivers start charging, *E* denotes the battery capacity, *β* denotes the electricity consumption rate, *d_average_* denotes the average daily distance traveled by an EV, and *σ* is the average daily charging times of an EV. Then, the demand for recharging each day can be expressed as $\sum_{i\in I}\tau D_{i}\sigma$, where *D_i_* is the car ownership of demand node *i* and *τ* is the penetration rate of the EV.

There are charging peaks during the weekday commute [54]. The government may implement a time-of-use tariff-setting method to relieve the pressure on the grid during peak periods. By the price elasticity coefficient, the ratio of charging during the peak periods can be estimated as $p_{re}=p_{r}+p_{r}e(\frac{c_{r}-c_{l}}{c_{r}})$, where *p_r_* is the percentage of charging during the peak periods, *e* is the price elasticity coefficient, *c_r_* is the charging price during the peak periods, and *c_l_* is the charging price during the off-peak periods. Then, the ratio of charging during the off-peak periods is $p_{le}=1-p_{re}$.

#### 2.4.2. Waiting Time and Idle Rate

The unreasonable location of charging stations will cause congestion and idleness [25], and the congestion often occurs during the peak charging periods, while idleness usually occurs during the off-peak periods. Therefore, we introduce waiting times during peak periods and the idle rate during off-peak periods to describe the service process. Let the charging power of a fast charger be *V_power_*. Then, the charging time can be expressed as:

(2) $$t_{charging}=\frac{(L_{soc}-S_{soc})E}{V_{\mathrm{power}}}.$$

EV drivers will give priority to the reservation service and arrive in an orderly manner without affecting others. The number of EVs using the reservation service can be expressed as $b_{ja}=a*\mu_{2}*(t_{l}+t_{r})$ where *μ*_2_ is the service rate of one reserved charging pile, *t_l_* is the off-peak duration, and *t_r_* is the peak duration. Then, the arrival rate during the peak periods can be expressed as:

(3) $$\lambda_{jr}=\frac{\sum_{i\in I}(x_{ij}\tau D_{i}\sigma -b_{ja})p_{re}}{t_{r}},$$

where *x_ij_* are the binary variables used to determine whether EVs in demand nodes *i* choose to be charged in station *j*. Then, the arrival rate during the off-peak periods can be expressed as:

(4) $$\lambda_{jl}=\frac{\sum_{i\in I}(x_{ij}\tau D_{i}\sigma -b_{ja})p_{le}}{t_{l}}.$$

Let the total number of charging piles at EVCS *j* be *h_j_*, and the number of charging piles for the reservation service is *a*. Then, the number of charging piles that are not being reserved is $s_{j}=h_{j}-a$.

According to the queueing theory, the M/M/s queueing system can be modeled as a birth-and-death process [48]. The equilibrium Equations (5) and (6) are satisfied when the system is at steady state.

(5) $$\lambda_{0}\pi_{0}=\mu_{1}\pi_{1}.$$

(6) $$\lambda_{n-1}\pi_{n-1}+\mu_{n+1}\pi_{n+1}=\pi_{n}(\lambda_{n}+\mu_{n})$$

where *λ_n_* is the birth rate at state *n*, *μ_n_* is the death rate at state *n*, and *π_n_* is the steady state probability at state *n*.

The supply–demand relationship of charging station services can be described by the service intensity. In this paper, the service intensity of the charging station during the off-peak periods can be expressed as:

(7) $$\rho_{jl}=\frac{\lambda_{jl}}{\mu_{1}s_{j}},$$

where *μ*_1_ is the service rate of one unreserved charging pile.

By combining the birth-and-death process with the difference equation, the steady state probability under steady state can be calculated as follows:

(8) $$\pi_{\mathrm{jl}0}={\left[\sum_{n=0}^{s_{j}-1}\frac{{\left(\rho_{jl}s_{j}\right)}^{n}}{n!}+\frac{{\left(\rho_{jl}s_{j}\right)}^{s_{j}}}{s_{j}!(1-\rho_{jl})}\right]}^{-1}.$$

(9) $$\pi_{j\ln}=\{\begin{array}{l} \frac{{\left(s_{j}\rho_{jl}\right)}^{n}\pi_{jl0}}{n!},n=1,2,\cdots ,s_{j}-1\\ \frac{{\left(s_{j}\rho_{jl}\right)}^{n}\pi_{jl0}}{s_{j}!s_{j}^{n-s_{j}}},n=s_{j},s_{j}+1,\cdots ,h_{j} \end{array}$$

The waiting time refers to the time from the driver starting to queue up at the station to commencement of the charging service. The average waiting time during the off-peak periods can be expressed as:

(10) $$t_{jl}^{q}=\frac{s_{j}^{s_{j}}\rho_{jl}^{s_{j}+1}\pi_{jl0}}{\lambda_{jl}s_{j}!{\left(1-\rho_{jl}\right)}^{2}}.$$

When the number of drivers in the charging station service system is less than the number of charging piles, the number of working charging piles is equal to the number of drivers. If the number of drivers in the charging station service system is larger than or equal to the number of charging piles, all charging piles are working. Then, the average number of working charging piles can be expressed as:

(11) $$\beta_{j}=\sum_{n=0}^{s_{j}-1}n\pi_{j\ln}+\sum_{n=s_{j}}^{\infty }s_{j}\pi_{j\ln},$$

The idle rate refers to the proportion of charging piles that are not working. In this paper, drivers will give priority to the reservation service, so the charging piles for the reservation service will always be working. Then, the average idle rate during the off-peak periods can be expressed as:

(12) $$f_{jl}=1-\frac{\beta_{j}+a}{h_{j}}.$$

Similar to the derivation of waiting time during off-peak periods, the average waiting time during peak periods can be expressed as:

(13) $$t_{jl}^{q}=\frac{s_{j}^{s_{j}}\rho_{jl}^{s_{j}+1}\pi_{jl0}}{\lambda_{jl}s_{j}!{\left(1-\rho_{jl}\right)}^{2}},$$

where

(14) $$\pi_{\mathrm{jl}0}={\left[\sum_{n=0}^{s_{j}-1}\frac{{\left(\rho_{jl}s_{j}\right)}^{n}}{n!}+\frac{{\left(\rho_{jl}s_{j}\right)}^{s_{j}}}{s_{j}!(1-\rho_{jl})}\right]}^{-1}.$$

#### 2.4.3. Objective for the Government

The purpose of building EVCSs, for the government, is to minimize the social cost, as EVs are currently in the promotion stage in China. The total social cost includes annual time opportunity cost, the annual traveling cost, the annual construction cost, and the annual operating cost [27].

The annual time opportunity cost for drivers is equal to the waiting time in the queue multiplied by the opportunity cost per unit of time, i.e., *c_c_*. The drivers who have a reservation do not need to wait for the introduction of the reservation service, and the waiting time during peak periods is different from that during off-peak periods, so the annual waiting time cost can be expressed as:

(15) $$z_{1}=365c_{c}\sum_{j\in J}[\sum_{i\in I}\left(x_{ij}\tau D_{i}\sigma -b_{ja})(p_{re}t_{jr}^{q}+p_{le}t_{jl}^{q})\right].$$

Annual traveling cost refers to the total traveling cost of all EVs that drive from demand nodes to charging stations to recharge the battery and is calculated each year.

(16) $$z_{2}=365\sum_{i\in I}\sum_{j\in J}c_{f}d_{ij}\tau D_{i}x_{ij}\sigma ,$$

where *c_f_* is the unit traveling cost and *d_ij_* is the distance from demand node *i* to charging station location *j*.

The annual construction cost of charging stations is based on an equivalent annuity. Let the basic construction cost for installing one charging pile be *c_v_*. Then, $\sum_{j\in J}c_{v}h_{j}$ is the total initial construction cost, including installation cost, purchasing charging piles cost, land cost, and the cost of other devices. The annual construction cost of charging stations can be expressed as:

(17) $$z_{3}=\frac{r_{0}{\left(1+r_{0}\right)}^{w}}{{\left(1+r_{0}\right)}^{w}-1}\sum_{j\in J}c_{v}h_{j},$$

where *r*_0_ is the discount rate and *w* is the investment period.

Annual operating cost is equal to the initial investment multiplied by the conversion factor (i.e., *ς*), including staff salaries, maintenance expense, and equipment depreciation expense [55].

(18) $$z_{4}=\varsigma \sum_{j\in J}c_{v}h_{j}$$

Therefore, to minimize the total social cost of the EVCS location planning, the objective for the model can be expressed as:

(19) $$\mathrm{Min}z=z_{1}+z_{2}+z_{3}+z_{4}$$

#### 2.4.4. Constraints

The long charging time of EVs compared with traditional cars may lead to congestion during peak periods. To improve the drivers’ satisfaction, the average waiting time during peak periods at each EVCS must be no more than the tolerable waiting time (i.e., *t_θ_*):

(20) $$t_{jr}^{q}\leq t_{\theta },j\in J$$

If we build too many charging stations to avoid congestion, it may cause many charging piles to be idle during the off-peak periods. To avoid investment waste, the average idle rate during off-peak periods at each EVCS must be no more than the tolerable idle rate (i.e., *f_θ_*):

(21) $$f_{jl}\leq f_{\theta },j\in J$$

The large-scale EV charging power will greatly impact the distribution network operation due to the uncertainty of the EV charging load [56]. For example. a large number of charging piles working at the same time may cause a voltage drop of the distribution network. To maintain the stability of the power grid, the number of charging piles built at each EVCS must be less than the number (i.e., *m*) of charging piles that a charging station allows being installed, which depends on the local power grid:

(22) $$h_{j}\leq m,\overset{}{}j\in J,$$

The service capacity of the charging station is limited, so the number of EVs recharged at station *j* must be no more than the service capacity of station *j*:

(23) $$\sum_{i\in I}x_{ij}A_{i}\tau \leq 24\mu_{1}h_{j},j\in J$$

EVs can be charged and charging piles can be installed in *j* only when there is a charging station located in *j*:

(24) $$x_{ij}\leq My_{j},\overset{}{}\;i\in I,j\in J,$$

where *M* is a large positive number and *y_j_* is the binary variables used to determine whether alternative charging station location *j* is selected.

A charging station can only provide service for EVs within its service range (i.e., *d*_max_):

(25) $$x_{ij}(d_{\max}-d_{ij})\geq 0,\overset{}{}\;i\in I,j\in J$$

To ensure that all EVs can be charged, the number of EVs needing to charge every day must equal the number of EVs that have been recharged.

(26) $$\sum_{j\in J}\;\sum_{i\in I}x_{ij}A_{i}\sigma =\sum_{i\in I}A_{i}\sigma$$

#### 2.4.5. Global Model

With increasingly serious environmental pollution, EVs have become an important way to develop sustainable transportation because of their advantages of zero-emission [49]. The governments of many countries have introduced various incentive policies to accelerate EVs adoption, intending to build sustainable transport systems [57]. This paper establishes a charging station location model aiming at minimizing the social cost from the perspective of the government. Due to the long charging time of EVs, the charging stations tend to be congested. Therefore, a reservation service is introduced to avoid long waiting times and improve system efficiency and customer satisfaction. Meanwhile, to improve the utilization of resources, the queuing system is introduced to describe the service process and establish the waiting time and idle rate constraints. Direct current charging is generally adopted for fast charging piles. The large-scale EV charging power will greatly impact the distribution network operation due to the uncertainty of the EV charging load [56]. Therefore, the number of charging piles installed at each charging station is restricted in this study. The global model can be expressed as follows:

(27) $$\mathrm{Min}\;z=z_{1}+z_{2}+z_{3}+z_{4}\;\begin{array}{c} st.\left\{\begin{array}{c} f_{jl}\leq f_{\theta },j\in J\\ t_{jr}^{q}\leq t_{\theta },j\in J\\ h_{j}\leq m,j\in J\\ \sum_{i\in I}x_{ij}A_{i}\tau \leq 24\mu_{1}h_{j},j\in J\\ x_{ij}\leq My_{j},\overset{}{}\;i\in I,j\in J\\ x_{ij}(d_{\max}-d_{ij})\geq 0,\overset{}{}\;i\in I,j\in J\\ \sum_{j\in J}\;\sum_{i\in I}x_{ij}A_{i}\sigma =\sum_{i\in I}A_{i}\sigma \end{array}\right. \end{array}$$

## 3. Results

**Figure 3:**
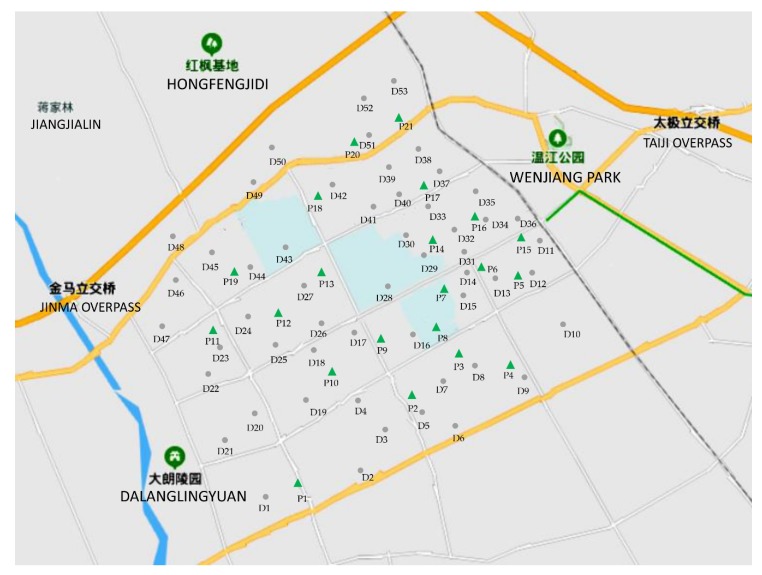
The urban area map of Wenjiang, Chengdu, China.

To verify the feasibility and effectiveness of the proposed method in this paper, the actual road system in the urban area of Wenjiang, Chengdu, China, was used. This area is approximately 36 km^2^, and the number of cars is about 18,061. By analyzing the distribution of cars and the road system, 53 demand nodes were identified and the number of cars at each demand node was estimated, as shown in Table 2. Parking lots could be transformed into centralized quick charging stations. Therefore, 21 parking lots in this area were taken as alternative charging stations, taking natural environment, geographical space, and other factors into consideration. The map is shown in Figure 3. The distances between demand nodes and alternative charging stations are the shortest traveling distances measured by the Baidu map tool, as shown in Table A1 of Appendix A.

Most charging events start with a 40%–50% SOC [52]. In this case study, we assume EV drivers start charging at a 40% SOC. Each vehicle travels 30 miles a day on average, the electricity consumption rate is 0.3 kWh/miles, and the battery capacity is 30 kWh [31]. The fast charger power is 60 kW [58], and we assume EV drivers leave the charging station at a 100% SOC, as discussed in Section 2. Then, according to Equations (1) and (2), we can calculate that each EV will be recharged once every two days (i.e., *σ* = 0.5), and the charging time will be about 18 min (i.e., *t_charging_* = 18 min). Considering that a series of actions during the charging service will take about 2 min [59], we assume that it takes drivers an average of 20 min from when they start receiving the service to when they leave the charging station, so the service rate is *μ*_1_ = 3. Then, to provide a buffer time for the users who have a reservation, the service rate is assumed as *μ*_2_ = 2.5.

The construction of charging stations in the urban area of Wenjiang to provide enough electric power for EVs requires a reasonable selection of some necessary parameters. The parameters were set based on previous research [54,55,60], and the specific values are shown in Table 3.

**Figure 4:**
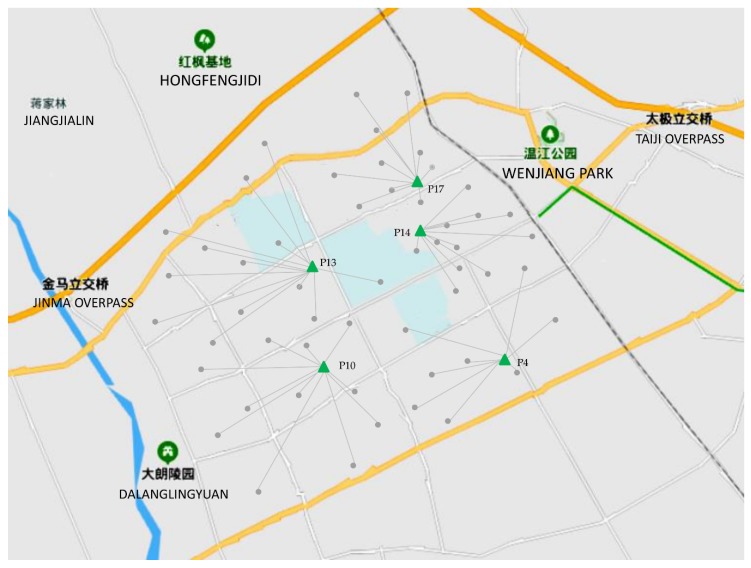
The locations of charging stations and their service areas.

To reduce the solution difficulty and avoid a situation where there is only one charging pile in a charging station, this study assumed that six charging piles could be constructed at each EVCS. The proposed model was solved by the genetic algorithm (GA), and the algorithm was coded in Matlab 2017b. The obtained optimal solution is shown in Table 4 and Table 5 and Figure 4. Under the assumptions of the model, it can be seen from Table 3 that when *τ* = 0.1, *a* = 0.1, and *m* = 6, five charging stations need to be built in this area at a total annual cost of 6,761,684 CNY.

## 4. Discussion

### 4.1. Benefit of the Reservation Service

To explore the effect of the reservation service on the optimal EVCS location, the proposed model was solved by the GA with a changing number of charging piles for a reservation service under different penetration rates. To analyze the advantages of the reservation service, the cases where the reservation service is not provided are also considered, i.e., *a* = 0.

**Figure 5:**
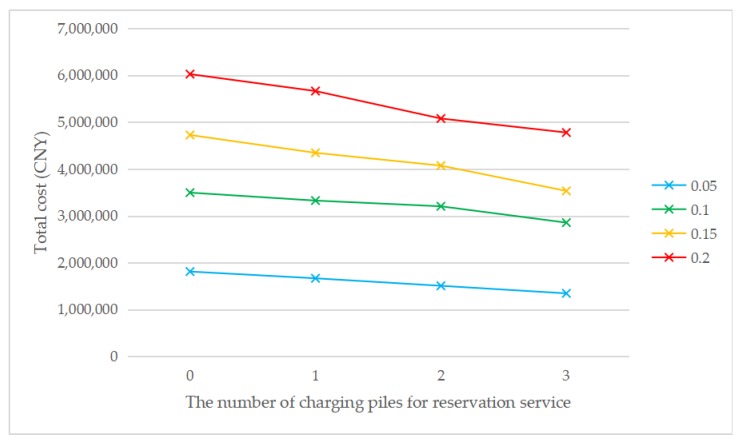
The total cost of changing the number of charging piles for the reservation service under different penetration rates.

**Figure 6:**
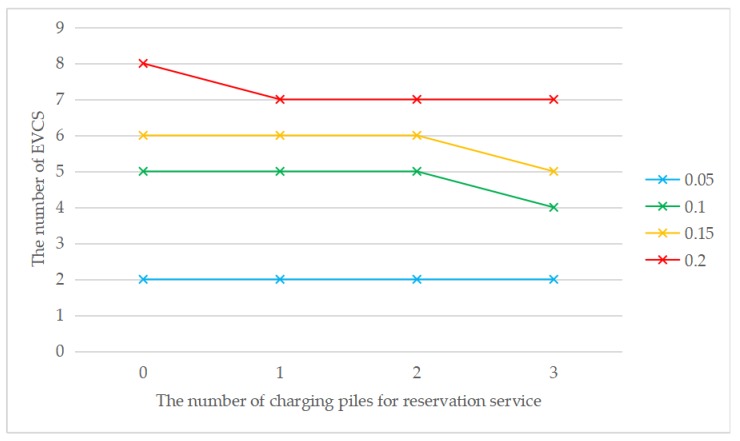
The number of EVCS with changing the number of charging piles for the reservation service under different penetration rates.

As shown in Figure 5, when the penetration rate is the same, the cost without considering the reservation service is always higher than the cost considering the reservation service, which means that the introduction of the reservation service has a positive impact on the EVCS location problem. Meanwhile, with the increase in the number of charging piles providing the reservation service, the total cost gradually decreases. The total cost always increases with the increase of penetration rate, regardless of the number of charging piles for the reservation service. This is consistent with reality, as the total number of chargers will increase to meet the increasing charging demand due to the increase in the total number of EVs. The construction cost is the main component in the cost structure. Figure 6 shows that the introduction of reservation services may reduce the number of EVCS needed. It can be seen that when *τ* = 0.1, *τ* = 0.15, or *τ* = 0.2, the introduction of the reservation service will reduce the number of EVCS required but not when *τ* = 0.05. It is also consistent with reality, because the introduction of the reservation service mainly affects the waiting time constraint, and when the penetration rate is low, the waiting time constraint is weak. When the penetration rate increases, the waiting time constraint is stronger, and the reservation service is more effective. The above analysis indicates that the introduction of the reservation service plays a positive role in reducing the total cost and improving social benefits. Meanwhile, the greater the penetration rate is, the more obvious the advantages of the reservation service will be.

**Figure 7:**
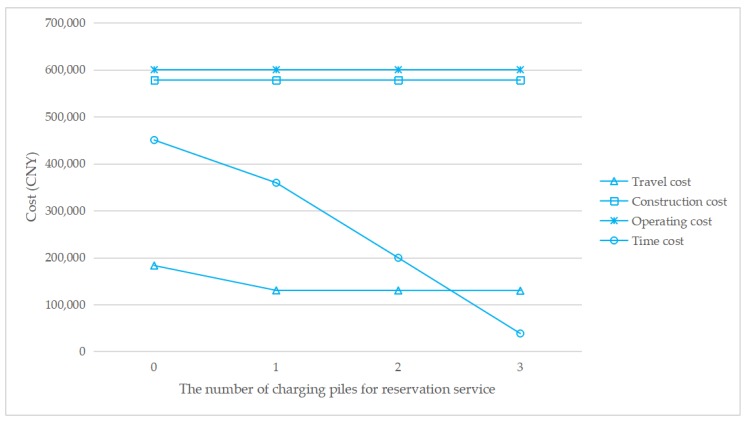
Cost change with a changing number of charging piles for the reservation service under *τ* = 0.05

**Figure 8:**
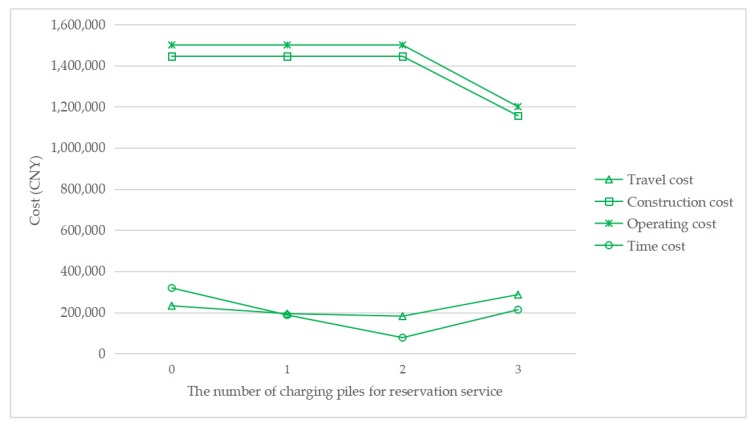
Cost change with a changing number of charging piles for the reservation service under *τ* = 0.1.

**Figure 9:**
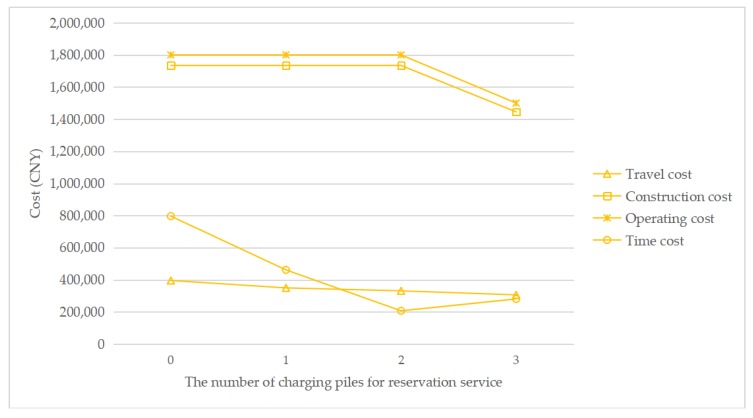
Cost change with a changing number of charging piles for the reservation service under *τ* = 0.15.

**Figure 10:**
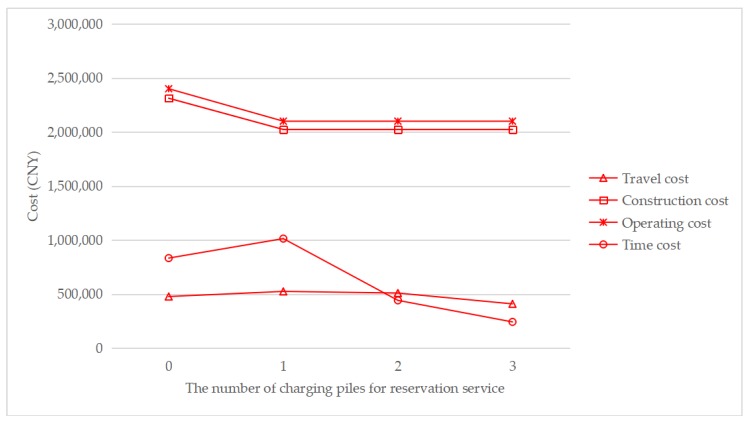
Cost change with a changing number of charging piles for the reservation service under *τ* = 0.2.

To understand how the reservation service affects the total cost, Figure 7, Figure 8, Figure 9 and Figure 10 show the cost structure with a changing number of charging piles for the reservation service under different penetration rates. It can be seen that the construction cost curve is always parallel to the operating cost curve, because in this paper, there is a linear conversion factor between the construction cost and the operating cost. As shown in Figure 7, when *τ* = 0.05, with the increase in the number of charging piles providing reservation services, the waiting time significantly decreases, and the driving cost also slightly decreases. However, when *τ* = 0.1, the number of charging piles providing the reservation service increases from 2 to 3, and the cost of waiting time and driving increases. A similar situation happens when *τ* = 0.15 and *τ* = 0.2. This seems unreasonable because drivers who have a reservation do not need to wait. However, Figure 6 shows that this unusual situation can be clearly explained. When *τ* = 0.1 and *a* = 3, the number of EVCS planned to be built is less than that under *τ* = 0.1 and *a* = 2, and as the number of EVCS decrease, the overall service capacity of the region decreases, resulting in an increase in waiting time.

In theoretical research, the impact of reservation services has been considered in many queuing scenarios [61,62]. Long queues for charging have also been one of the important factors hindering the development of the electric car industry. However, in the area of EVCS location, few people have considered the impact of a reservation service. Therefore, this paper introduces a reservation service into the EVCS location problem. Through a comparative analysis, it can be found that the introduction of a reservation service has a positive impact, as it can reduce the total cost compared with no reservation service. The more charging piles for the reservation service, the less it costs. Meanwhile, the larger the EV penetration rate, the more obvious the advantages of the reservation service.

In reality, the introduction of a reservation service also conforms to the development of a smart city. The drivers can make a reservation in advance, so that they can better plan their trips and make them more comfortable and convenient. Meanwhile, EVs are at the promotion stage, and there are few EVCSs. Some stations may only be known by a small number of drivers. By introducing the reservation service, the driver will know the location of the EVCS and the number of available charging piles, and will generally choose the charging station with free charging piles within an acceptable distance, so as to improve the utilization rate of resources.

The goal of the government is to minimize the total social cost of EVCS locations, as EVs are in the promotion stage in China. However, in some scenarios, considering the impact of reservation services when selecting EVCS sites can lead to a longer time for drivers traveling to charging stations, because sometimes the increase in reservation service capacity reduces the number of EVCS that need to be built but increases the time cost. Therefore, in reality, there is a need to balance the construction cost and drivers’ tolerable waiting time.

### 4.2. Effect of Tolerable Waiting Time

**Figure 11:**
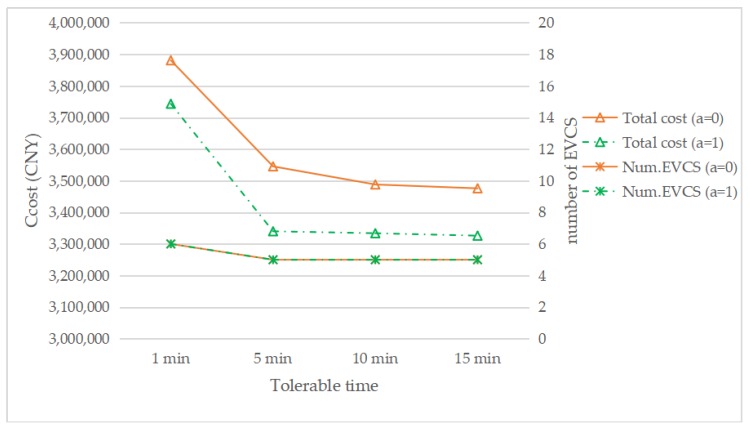
The total cost and EVCS number with changing the tolerable time.

The effect of different tolerable waiting times was examined in this study. The tolerable waiting time (i.e. *t_θ_*,) was assumed to be 15 min in the above discussions. In addition to the case for 15 min, the cases for 1, 5, and 10 min when *τ* = 0.1, *a* = 1, or *a* = 0 respectively, are also discussed here. It can be seen from Figure 11 that with a decrease in tolerable time, the total cost increases. In addition, when the waiting time is reduced from 5 to 1 min, the suggested number of EVCS to be built increases by one. Reduced tolerable waiting times mean fewer EVs can be served at each EVCS, which will increase the number of EVCS required. Meanwhile, Figure 11 shows that when the tolerable time is the same, the total cost with the reservation service is always less than that without the reservation service.

**Figure 12:**
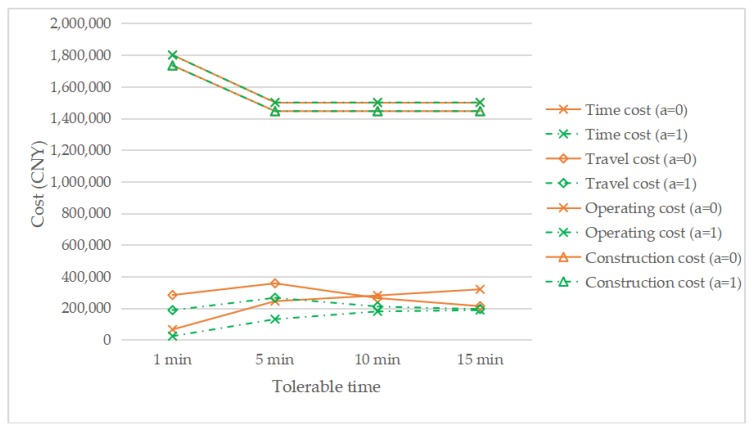
The change of cost components with changing the tolerable time.

Figure 12 shows the changing cost structure with the change in the tolerable time under *τ* = 0.1, *a* = 1, and *a* = 0. The construction cost and operating cost of *a* = 1 are equal to that of *a* = 0, because the number of EVCS required is the same, which can be seen from Figure 11. It can be seen that, with a decrease in tolerable time, both time cost and travel cost increase. In addition, as the tolerable time decreases, the difference between the time cost curve with the reservation service and the time cost curve without the reservation service also increases, which means that the more that people are averse to waiting, the more effective the reservation service will be. Compared with the traditional car, the longer charging time of electric vehicles is one of the factors that hinder the development of EVs. However, if there is congestion in the charging station, drivers in the queue must wait at least 20 min under the assumption in this paper, which will undoubtedly further reduce the acceptance of EVs by drivers, thus further hindering the development of the EV industry. Therefore, the introduction of a reservation service can reduce the waiting time of the driver who made the reservation to 0, so that drivers can have a better, more convenient plan, and feel more comfortable, which will improve drivers’ satisfaction and promote the development of EVs.

Based on existing related literature, this study introduces a reservation service into the EVCS location problem. The analysis above shows that the introduction of a reservation service has a positive impact on this problem, as it can reduce the total social cost and enhance customer convenience by reducing the waiting time compared with no reservation service.

## 5. Conclusions

Based on the development of the Internet, this paper introduced a reservation service into the EVCS location problem for the development of sustainable cities. Meanwhile, considering the long charging time of EVs, there will be congestion during the peak periods and idle equipment during the off-peak periods. Therefore, this paper introduced the constraint of average queuing time during the peak periods and idle rate during the off-peak periods to balance the service and improve resource utilization. Finally, a case from Chengdu, China was considered to test the feasibility of the proposed model. The results show that the introduction of a reservation service can not only reduce the total social cost, but also lower the waiting time of users, resulting in increased convenience for customers.

The contributions of this study can be summarized as follows. First, a reservation service is introduced into the EVCS location problem for reducing waiting times at the charging stations. Second, a model for the location problem allowing for a reservation service is proposed to minimize the total social cost. Moreover, analyses of results obtained using the proposed model are performed. These analyses shed light on the effects of different penetration rates and tolerable waiting times on the EVCS location planning. Compared to previous literature without a reservation service, the main merit of this study is that the introduction of the reservation service can not only benefit the drivers by reducing waiting time cost, but also benefit other stakeholders by decreasing the total social cost, which is of positive significance for the promotion of EVs towards sustainable cities for healthier lives.

There are a few limitations of this study. First, the characteristics of user charging behavior were not fully considered. Moreover, this paper considers technology-related parameters (e.g., service rates) as fixed values, but technologies may be improved in the future. Future research will focus on the following three directions: (1) fully considering the characteristics of user charging behavior with regard to the EVCS location problem, (2) integrating management optimization and technical innovation to improve the EVCS location, and (3) developing comprehensive planning for sustainable EVCS management systems.

## Figures and Tables

**Table 1:** Notations used in this study.

Sets	Description
*I*	The set of demand nodes.
*J*	The set of alternative charging station locations.
**Parameters**	**Description**
*D_i_*	The number of cars at demand nodes *i*.
*τ*	The penetration rate of EVs.
*C_r_*	The charging price during the peak periods.
*C_l_*	The charging price during the off-peak periods.
*λ_jr_*	The arrival rate during the peak periods.
*λ_jl_*	The arrival rate during the off-peak periods.
*P_r_*	The percentage of charging during the peak periods.
*P_re_*	The percentage of charging after a price adjustment during the peak periods.
*P_le_*	The percentage of charging after a price adjustment during the off-peak periods.
*μ*_1_	The service rate of one unreserved charging pile.
*μ*_2_	The service rate of one reserved charging pile.
*C_c_*	The time opportunity cost (CNY/h).
*C_o_*	The annual operating cost (CNY/year).
*C_v_*	The basic construction cost for installing one charging pile.
*C_f_*	The unit traveling cost (CNY/km).
*ς*	The conversion factor.
$t_{jr}^{q}$	The average waiting time during the peak periods (h).
*t_θ_*	The tolerable waiting time.
*f_jl_*	The average idle rate.
*f_θ_*	The tolerable idle rate.
*w*	The investment periods (year).
*π_j_*_ln_	The probability of n EVs in the queuing system during the peak periods.
*π_j_*_rn_	The probability of n EVs in the queuing system during the off-peak periods.
*a*	The number of charging piles for the reservation service.
*b_ja_*	The number of reserved EVs.
*s_j_*	The number of charging piles that are not being reserved.
*t_r_*	The peak duration (h).
*t_l_*	The off-peak duration (h).
*r_0_*	The discount rate (%).
*d_ij_*	The distance from demand node *i* to charging station location *j* (km).
*d_max_*	The tolerable distance (km).
*e*	The elastic coefficient.
*M*	A large positive number.
*m*	The number of charging piles that a charging station allows to install.
$t_{jl}^{q}$	The average waiting time during the off-peak periods (h).
**Decision Variables**	**Description**
*y_j_*	The binary variables used to determine whether alternative charging station location *j* is selected.
*x_ij_*	The binary variables used to determine whether EVs in demand node *i* choose to be charged in station *j*.
*h_j_*	The number of charging piles that are installed in alternative charging stations location *j*.

**Table 2:** The number of cars at each demand node.

Demand Nodes	1	2	3	4	5	6	7	8	9
Number of Cars	237	337	278	384	330	251	490	330	419
Demand Nodes	10	11	12	13	14	15	16	17	18
Number of Cars	420	350	462	380	339	390	488	330	420
Demand Nodes	19	20	21	22	23	24	25	26	27
Number of Cars	266	222	283	240	220	239	267	440	400
Demand Nodes	28	29	30	31	32	33	34	35	36
Number of Cars	370	426	369	208	294	320	320	359	370
Demand Nodes	37	38	39	40	42	42	43	44	45
Number of Cars	420	330	390	340	450	430	270	276	306
Demand Nodes	46	47	48	49	50	51	52	53	/
Number of Cars	267	220	294	390	300	420	330	380	/

**Table 3:** The values of necessary parameters.

Parameters	Values	Parameters	Values
τ	0.1	$c_{c}$	30 CNY/h
$p_{r}$	51.3%	$c_{v}$	500,000 CNY/pile
$p_{l}$	48.7%	$c_{f}$	0.4 CNY/km
$\mu_{1}$	3	$\mu_{2}$	2.5
$t_{\theta }$	0.25 h (15 min)	a	1
$f_{\theta }$	0.7	$d_{\max}$	4 km
$t_{r}$	7.5 h (6:30–9:00, 16:00–21:00)	ς	0.1
$t_{l}$	16.5 h	$r_{0}$	5%
h	8	w	15
$c_{r}$	1.8 CNY/kWh	$c_{l}$	1.2 CNY/kWh
e	−0.08		

**Table 4:** The planning cost of the optimal solution.

Cost	Traveling	Annual Construction	Annual Operating	Annual Time Opportunity	Total Cost
Amount (CNY)	193,758	1,445,134	1,500,000	187,217	3,326,110

**Table 5:** The list of selected charging stations and corresponding service areas.

EVCS Number	Service Area
4	(5), (6), (7), (8), (9), (10), (12), (16)
10	(1), (2), (3), (4), (17), (18), (19), (20), (21), (22), (25)
13	(23), (24), (26), (27), (28), (43), (44), (45), (46), (47), (48), (49), (50)
14	(11), (12), (14), (15), (29), (30), (31), (32), (34), (35), (36)
17	(33), (37), (38), (39), (40), (41), (42), (51), (52), (53)

## Appendix A

Table A1 lists the distance between demand nodes and alternative charging stations.

**Table A1:** The distance between demand nodes and alternative charging stations (km).

	Demand Nodes	1	2	3	4	5	6	7	8	9	10	11	12	13	14	15	16	17	18	19	20	21
Charging Stations																						
1		1.466	3.332	4.283	4.803	6.023	6.146	5.851	4.928	4.027	3.325	3.658	4.378	4.998	7.022	6.655	7.053	7.140	6.256	4.285	7.907	8.776
2		2.031	1.662	2.763	3.819	4.269	4.626	4.331	3.408	2.507	1.805	3.877	2.906	3.526	5.526	5.135	5.533	5.644	4.736	4.056	6.411	7.280
3		2.179	1.127	1.733	2.338	3.860	3.596	3.301	2.378	1.477	1.548	3.620	2.649	2.996	4.496	4.105	4.503	4.614	3.706	4.247	5.381	6.250
4		2.355	1.662	2.268	3.552	3.682	3.418	3.123	2.831	1.299	0.941	2.928	2.042	2.818	4.318	3.927	4.325	4.436	3.528	3.192	5.203	6.072
5		2.696	0.236	1.460	1.896	3.116	3.323	3.028	2.105	1.204	2.065	4.137	3.201	2.723	4.223	3.832	4.230	4.341	3.433	4.351	5.108	5.977
6		3.128	1.629	1.945	2.125	3.345	3.348	3.551	2.590	2.512	3.373	5.320	4.509	4.031	4.486	4.355	4.753	5.649	4.741	5.947	6.416	7.285
7		2.972	1.130	1.093	1.850	3.258	2.994	2.699	1.738	1.480	2.341	4.413	3.477	2.999	3.634	3.503	3.901	4.617	3.709	5.040	5.384	6.253
8		3.380	1.538	0.195	1.207	2.427	2.430	2.553	1.126	1.888	2.749	4.821	3.885	3.407	3.488	3.357	3.755	3.906	4.117	5.448	4.673	5.542
9		5.070	2.077	1.458	0.436	1.365	1.368	2.007	2.418	3.155	3.999	6.071	5.152	4.674	2.605	2.332	2.730	3.042	4.342	6.698	3.809	4.519
10		5.447	2.894	2.275	1.253	1.723	2.501	3.140	3.551	3.972	4.816	6.888	5.599	5.121	3.753	1.906	2.943	4.182	5.475	6.749	4.949	5.445
11		5.934	4.092	3.538	2.961	1.404	1.324	1.963	2.374	3.417	3.872	5.593	4.422	3.944	2.576	0.729	1.766	3.005	4.298	5.572	3.772	4.268
12		5.772	3.930	3.376	1.970	0.518	1.162	1.801	2.212	3.255	3.710	5.431	4.260	3.782	2.414	1.206	1.604	2.843	4.136	5.410	3.610	4.040
13		5.006	3.164	2.610	1.890	0.903	0.396	1.035	1.446	2.489	2.944	4.665	3.494	3.016	1.662	0.905	1.303	2.070	3.370	4.644	2.837	3.739
14		5.087	3.245	2.691	1.971	1.196	0.464	0.879	1.527	2.570	3.025	4.162	2.911	2.433	0.852	1.441	0.983	1.289	2.787	4.061	2.048	2.766
15		4.474	2.632	2.078	1.837	1.062	0.798	0.503	0.914	1.957	2.412	4.133	2.962	2.484	1.438	1.307	1.705	1.856	2.838	4.112	2.623	3.492
16		3.163	1.321	1.586	2.854	2.169	1.905	1.610	1.318	0.958	1.606	3.327	2.563	2.085	2.545	2.414	2.812	2.963	2.795	3.713	3.730	4.599
17		3.359	1.517	2.123	3.407	2.640	2.376	2.081	1.789	0.842	1.066	2.787	1.842	0.916	2.864	2.885	3.283	2.982	2.074	2.992	3.749	4.618
18		3.240	2.886	3.492	4.760	3.969	3.705	3.565	3.118	2.171	1.002	1.854	1.069	1.689	3.713	4.214	4.379	3.831	2.923	2.219	4.598	5.467
19		2.004	2.428	3.034	4.302	3.511	3.247	2.952	2.660	1.713	0.544	1.518	1.419	1.965	3.887	3.756	4.154	4.265	3.357	2.145	5.032	5.901
20		1.374	2.834	3.440	4.708	4.161	3.897	3.602	3.310	2.363	1.194	1.258	1.978	2.598	4.622	4.406	4.804	4.740	3.832	1.885	5.507	6.376
21		1.805	3.265	3.871	5.139	5.566	5.302	5.007	4.516	3.121	2.372	2.663	3.383	4.003	6.027	5.811	6.209	6.145	5.237	3.290	6.912	7.781
22		3.186	4.646	5.252	6.520	5.538	5.274	5.016	4.687	3.740	2.571	1.800	2.520	3.140	5.164	5.783	5.830	5.282	4.374	2.427	6.049	6.918
23		2.599	4.059	4.665	5.933	5.087	4.565	3.856	4.165	3.218	1.984	0.165	1.360	1.980	4.004	5.332	4.670	4.122	3.214	1.267	4.889	5.758
24		2.860	4.284	4.926	6.194	4.990	4.468	3.759	4.556	3.609	2.367	0.901	1.263	1.883	3.907	5.235	4.573	4.025	3.117	1.170	4.792	5.661
25		3.371	3.092	3.698	4.966	4.175	3.911	3.616	3.324	2.377	1.208	2.060	1.275	1.895	3.919	4.420	4.585	4.037	3.129	2.425	4.804	5.673
26		3.449	1.937	2.543	3.827	3.060	2.796	2.501	2.209	1.262	1.242	2.633	1.382	0.748	2.941	3.305	3.607	3.059	2.151	2.532	3.826	4.695
27		4.204	2.356	2.962	4.246	3.623	3.101	2.392	2.607	1.681	1.640	2.245	0.994	0.360	2.540	3.868	3.206	2.658	1.750	2.144	3.425	4.294
28		4.329	2.487	2.216	3.484	2.830	2.566	1.990	1.979	1.812	2.267	3.257	2.006	1.528	2.138	3.075	2.804	2.256	2.238	3.156	3.023	3.892
29		5.454	3.612	3.058	2.448	1.673	0.941	0.936	1.894	2.937	3.392	3.692	2.441	1.963	0.382	1.929	1.046	1.188	2.317	3.591	1.955	2.824
30		5.404	3.593	3.356	2.746	1.971	1.239	1.234	2.192	2.918	3.298	3.445	2.194	1.716	0.001	2.227	1.344	1.046	2.070	3.344	1.813	2.682
31		5.841	3.999	3.445	2.725	1.273	1.231	1.666	2.232	3.256	3.769	4.492	3.251	2.687	1.172	1.595	0.75	1.731	3.157	4.411	2.511	2.712
32		5.929	4.118	3.301	2.581	1.627	1.074	1.602	2.137	3.443	3.823	3.970	2.719	2.241	0.911	1.629	0.746	1.029	2.595	3.869	1.796	2.166
33		5.805	3.994	3.956	2.758	2.235	1.251	1.834	2.792	3.319	3.699	3.846	2.595	2.117	0.787	2.237	1.354	0.641	2.471	3.745	1.408	2.118
34		5.941	4.099	3.545	2.825	1.373	1.331	1.856	2.381	3.424	3.879	4.612	3.361	2.883	1.302	1.023	0.001	1.731	3.237	4.511	2.498	2.603
35		6.266	4.424	3.870	3.150	1.698	1.656	2.181	2.706	3.749	4.204	4.651	3.400	2.922	1.627	1.700	0.326	1.710	3.276	4.550	2.477	2.314
36		6.041	4.199	3.645	2.925	1.473	1.331	1.956	2.581	3.500	3.979	4.712	3.451	2.953	1.352	0.750	0.457	1.831	3.287	4.601	2.507	2.613
37		6.368	4.557	4.519	3.321	2.401	1.814	2.397	3.355	3.882	4.262	4.409	3.158	2.68	1.35	2.403	1.520	0.918	2.289	4.308	1.269	1.543
38		6.581	4.770	4.254	3.534	3.011	2.027	2.610	3.090	4.095	4.475	4.622	3.371	2.893	1.563	3.013	1.777	1.106	2.155	4.174	1.135	1.409
39		6.052	4.241	4.203	3.617	3.092	2.105	2.081	3.039	3.566	3.946	4.093	2.842	2.364	1.484	3.094	2.511	0.858	1.472	3.491	0.704	1.941
40		6.079	4.268	3.753	3.197	2.669	1.690	2.108	2.589	3.593	3.973	4.120	2.869	2.391	1.061	2.671	1.788	0.132	2.392	4.019	1.372	2.241
41		5.777	3.966	3.928	3.812	3.037	2.515	1.806	2.764	3.291	3.671	3.818	2.567	2.089	1.956	3.282	2.620	0.721	1.308	3.327	1.407	3.210
42		6.138	4.296	4.902	4.621	3.846	2.784	2.615	3.573	3.621	4.076	4.148	2.897	2.419	2.136	4.091	2.736	1.680	0.952	2.971	0.929	2.166
43		3.779	3.59	4.163	5.431	4.296	3.774	3.065	3.862	2.915	1.673	1.820	0.569	1.189	3.213	4.541	3.879	3.331	1.391	0.669	3.012	4.249
44		3.397	4.091	4.664	5.932	4.797	4.275	3.566	4.363	3.416	2.174	1.438	1.070	1.690	3.714	5.042	4.380	3.832	1.858	0.190	3.479	4.716
45		4.026	4.96	5.566	6.100	5.325	4.803	4.094	5.232	4.285	2.875	2.067	1.771	2.391	4.244	5.570	4.908	3.989	1.617	1.372	3.238	4.475
46		3.326	4.786	5.392	6.660	5.456	4.934	4.225	5.022	4.075	2.711	1.367	1.729	2.349	4.373	5.701	5.039	4.491	2.092	0.672	3.713	4.95
47		4.209	5.669	6.275	7.543	6.339	5.817	5.108	5.905	4.958	3.594	2.250	2.612	3.232	5.256	6.584	5.922	5.119	2.747	1.555	4.368	5.605
48		3.943	5.403	6.009	6.373	5.598	5.076	4.367	5.505	4.558	3.148	1.984	2.044	2.966	4.517	5.843	5.181	4.262	1.890	1.289	3.511	4.748
49		4.721	4.150	4.756	5.290	4.515	3.993	3.284	4.422	3.475	2.615	2.762	1.511	2.273	3.434	4.760	4.098	3.179	0.807	1.577	2.428	3.665
50		5.512	3.670	4.276	4.700	3.925	3.403	2.694	3.942	2.995	3.450	3.522	2.271	1.793	2.844	4.17	3.508	2.589	0.327	2.366	1.838	3.075
51		6.924	5.113	4.598	4.039	3.514	2.527	2.953	3.434	4.438	4.818	4.965	3.714	3.236	1.906	3.516	2.506	1.450	2.087	4.106	0.583	1.936
52		6.823	4.981	5.587	3.946	3.421	2.434	2.860	3.341	4.306	4.761	4.833	3.582	3.104	1.813	3.423	2.413	1.357	1.637	3.656	0.001	1.843
53		7.477	5.635	4.802	4.111	3.219	2.604	3.157	3.638	4.960	5.022	5.169	3.918	3.440	2.110	3.221	2.226	1.654	2.291	4.310	1.271	1.211
